# *ITree*: a user-driven tool for interactive decision-making with classification trees

**DOI:** 10.1093/bioinformatics/btae273

**Published:** 2024-04-18

**Authors:** Hubert Sokołowski, Marcin Czajkowski, Anna Czajkowska, Krzysztof Jurczuk, Marek Kretowski

**Affiliations:** Faculty of Computer Science, Bialystok University of Technology, Bialystok 15-351, Poland; Faculty of Computer Science, Bialystok University of Technology, Bialystok 15-351, Poland; Department of Medical Biology, Medical University of Bialystok, Bialystok 15-089, Poland; Faculty of Computer Science, Bialystok University of Technology, Bialystok 15-351, Poland; Faculty of Computer Science, Bialystok University of Technology, Bialystok 15-351, Poland

## Abstract

**Motivation:**

ITree is an intuitive web tool for the manual, semi-automatic, and automatic induction of decision trees. It enables interactive modifications of tree structures and incorporates Relative Expression Analysis for detecting complex patterns in high-throughput molecular data. This makes ITree a versatile tool for both research and education in biomedical data analysis.

**Results:**

The tool allows users to instantly see the effects of modifications on decision trees, with updates to predictions and statistics displayed in real time, facilitating a deeper understanding of data classification processes.

**Availability and implementation:**

Available online at https://itree.wi.pb.edu.pl. Source code and documentation are hosted on GitHub at https://github.com/hsokolowski/iTree and in supplement.

## 1 Introduction

Decision trees (DTs) are one of the most popular interpretable machine learning models. The hierarchical structure of the tree, where appropriate tests are applied successively from one node to the next, closely resembles the human way of making decisions. However, in context of high-throughput biomedical data, traditional *DT*s have inferior prediction performance due to over and under-fitting issues, thus ensemble models like random forests or gradient-boosted trees are preferred. Unfortunately, these alternatives produce complex predictive models that impede logical reasoning and biological understanding patterns in the data.

The ease of visualizing the relationships between individual features is one of the main advantages of *DT*s prediction model. However, existing software primarily focuses on the static illustration of the induced DT, often only presenting its final form. The user has no influence on the appearance of the output tree aside from being able to set the basic parameters of the algorithm before running it like maximum depth, pruning, splitting strategy.

*ITree* is a web-based platform that enables users to interact with a visualized *DT* model created using a user-provided dataset. Once the training set is uploaded and the essential settings are configured, the platform builds the classification tree and displays the accuracy score and confusion matrix. Users can also upload a testing set to evaluate the prediction model’s performance and instance distribution. Decision tree models can be imported and exported in JSON format, allowing users to load pre-built trees generated with ITree or other systems, such as those from scikit-learn (Pedregosa *et al.* 2011).

The distinguishing characteristic of *ITree* is its capability to adjust and revise the prediction model. Users can alter the splits or suggest which attributes they want to use for partitioning a specific node. The top splits for each node are also presented to facilitate decision-making. The tree node can be partitioned by one level or fully expanded based on the chosen partitioning algorithm. Moreover, pruning any node down to a leaf is an option. Any changes made will be promptly reflected in the prediction accuracy statistics, confusion matrix, and testing dataset results.

*ITree* goes beyond using conventional univariate tests in the internal nodes. It uncovers hidden knowledge by adapting the Relative Expression Analysis (*RXA*) concept (Geman *et al.* 2004) to capture more advanced patterns between the features. *RXA* represents a simple yet powerful collection of easily interpretable classifiers, such as the “Top Scoring Pair” (Tan *et al.* 2005, Czajkowski 2023), which are designed to extract meaningful rules from high-dimensional biomedical data. As its idea is to compare between features relative expression levels within the same sample, the *ITree* with *RXA* splits may be robust to inter- and intra-platforms variabilities as well as complex analytical and data processing methods like normalization and standardization procedures (SungHwan *et al.* 2016).

## 2 Materials and methods

*ITree* induces a binary classification tree with the top-down greedy approach (Loh 2014) where locally best splits are made in each node. It starts from the root node, where the best split is searched. For node splitting, we adopted the Gain Ratio metric as the criterion, which is founded on entropy minimization. This metric is frequently utilized in *DT* algorithms, most notably in the *C4.5* algorithm, due to its effectiveness in achieving optimal splits by balancing the distribution of classes. Next, the training samples are redirected to the newly created subnodes and this process is repeated for each subnode until some stopping-rule is satisfied. The post-pruning is not applied after induction as it can be done manually by the user.

*ITree* allows three types of tests that split the nodes:

- *C4.5*-like splitting tests that use the concept of entropy, which measures the impurity or disorder in a set of instances. The attribute with the highest information gain is chosen as the splitting attribute. The splitting test has different approaches for various types of attributes. For continuous attributes, it seeks an optimal threshold value to divide the data into two subsets. In the case of nominal (discrete) attributes, the test examines each distinct attribute value. It then evaluates the information gain or gain ratio for these values when splitting the data. This univariate, axis-parallel split is based on a single attribute compared against a threshold value and has a form: attribute≥threshold value.
- Top-Scoring-Pair (*TSP*) test that seeks to identify typical reversals’ pair of genes/molecules (Geman *et al.* 2004). TSP test manifests as a comparison: $\mathrm{attribut}e_{1}<\mathrm{attribut}e_{2}$. This pairwise comparison split, devoid of linear attribute weighting, embodies a binary relational form. Within bioinformatics, its utility is unparalleled, spotlighting nuanced yet vital genomic patterns, like the identification of key markers or “biological switches” indicative of gene expression shifts or regulatory network alterations. The TSP’s methodological simplicity, interpretive transparency, and direct relevance to molecular data analysis render it an invaluable asset in uncovering the cryptic mechanisms underlying biological processes.
- Weight TSP (*WTSP*) test is a more advanced version of *TSP* and considers relative fraction comparisons between two attributes that constitute the top pair. (Czajkowski *et al.* 2020). This way we can detect not only the ordering shifts between the attributes but also the percent changes in their relations. This approach can be symbolically represented as: $\mathrm{attribut}e_{1}<\mathrm{weight}*\;\mathrm{attribut}e_{2},\;$where the *weight* factor adjusts the comparison to identify not only which feature is expressed more but also how much more it is expressed relative to another. Such weighted comparisons are crucial in bioinformatics applications, where the relative abundance of gene expressions can indicate critical biological processes or disease states. The WTSP test thus offers a more flexible and informative approach to understanding gene interactions, providing insights that are directly applicable to biomarker discovery and the elucidation of gene regulatory networks. However, it should be noted that it is much more calculation demanding than *TSP* or *C4.5.*

*ITree* is written entirely in JavaScript, and the tree construction, computations, and statistics are all performed on the user’s browser. This feature enables *ITree* to be used locally, even without internet access or as a plugin in larger machine learning-based decision support system. The application code is open and can easily be modified to perform the tree induction on the external server, which can be crucial when dealing with large datasets. Even during testing with thousands of attributes and objects, the *ITree* tool still functioned relatively quickly, operating entirely within the user’s web browser.

## 3 Results and highlighted features

**Figure 1.**
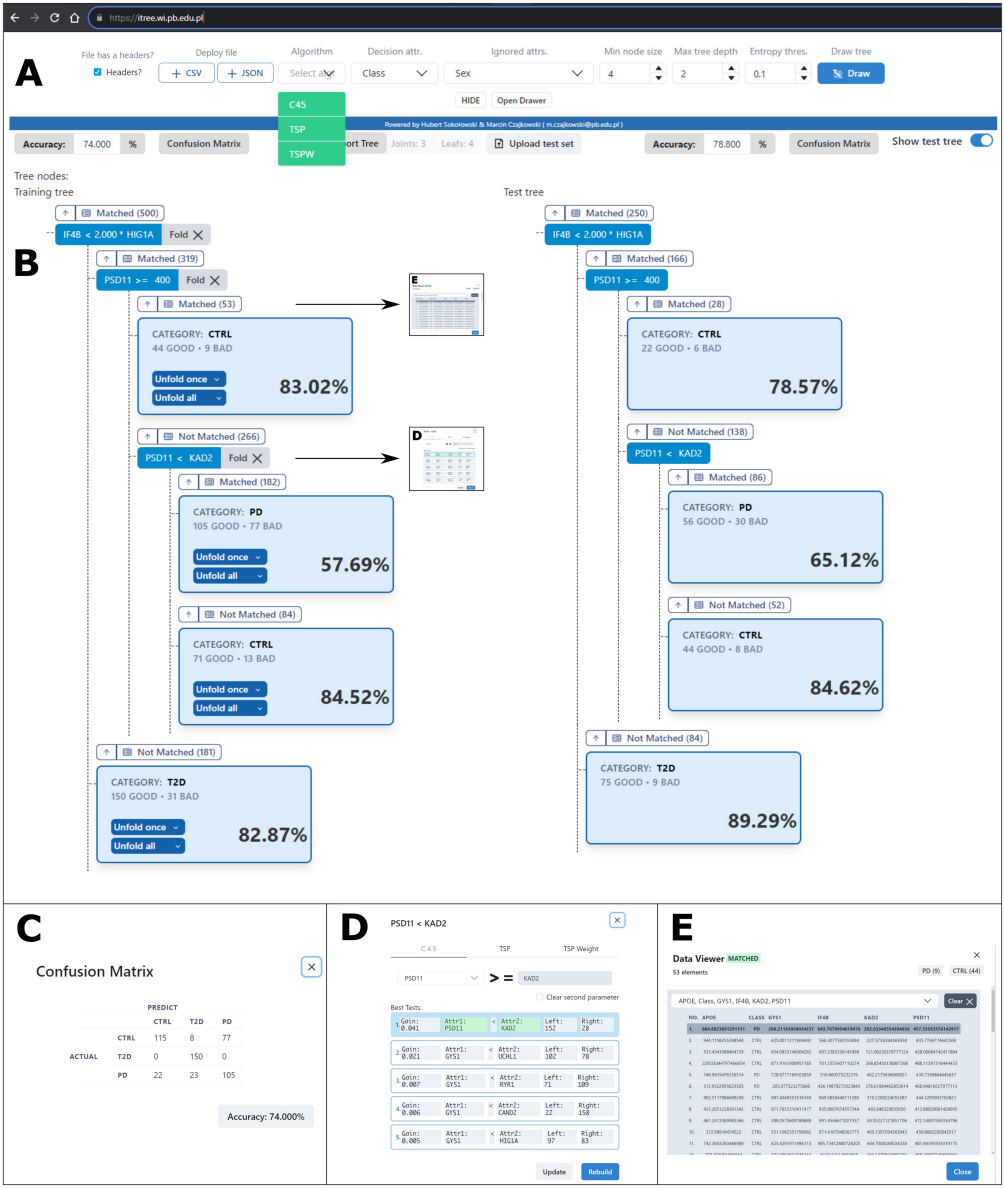
Illustration of ITree with an example experiment: (A) initial configuration of the Decision Tree (DT). (B) Visualization of the DT, showing training data (left) and testing data (right). (C) Display of the Confusion Matrix for the training dataset. (D) Options for test modifications and provided hints for one of the tree node splits. (E) Detailed information about selected instances that reached a particular node.

The *ITree* tool allows us to fully control the output of the Decision Tree (DT), its structure, as well as the induction process. In Fig. 1A and B, we present the main view of the *ITree* website, while the rest of the elements (C–E) are dialog windows that may appear based on user activity.

### 3.1 Interactive decision tree viewer

At the top of the *ITree* website is an initial configuration panel (Fig. 1A). After loading a training dataset in a standard CSV format, the user selects up to three split algorithms (*C4.5*, *TSP*, *WTSP*) and the decision attribute (class). Users can also choose attributes in the dataset to be ignored and modify a set of default *DT* parameters, such as minimum node size, maximum tree size, or the desired entropy threshold. Once the setup is complete, the “Draw” button activates, enabling *ITree* to generate the tree from the training set and offering options to upload the test set.

The central view of the *ITree* website is shown in Fig. 1B. The tree on the left, generated from the training set, is interactive. The root node or any internal tree node (represented as small rectangles) can be manually pruned to a leaf using the “Fold” option. All tree leaves (large, light-blue rectangles) hold information about the class label, the number of instances that reached the leaf, and overall classification accuracy. Each leaf can be expanded into a subtree.

Users can view the instances and select features that reached a specific node (Fig. 1E) and change the splitting rules by simply clicking on the tests (Fig. 1D). In the example shown, there is a *WTSP* test at the root, a *C4.5* split in the following node, and finally, a *TSP* relation is analyzed in the lowest part of the tree. Any changes made to the tree automatically trigger a recalculation of the tree’s statistics, such as accuracy and confusion matrix (Fig. 1C). At the same time, the view of the DT generated for the test set (tree on the right of Fig. 1B) is also refreshed.

### 3.2 Manual tests adjustments

One of the most important features of *ITree* is the ability to manually intervene in an already built *DT*. In practical applications, users frequently encounter diverse issues that they might need to address or correct in the output model. To the best of our knowledge, ITree is the first system that allows for these modifications on the fly. Usually the user-preferred features must be assigned ad-hoc as well as any cost-sensitive classification parameters. However, *ITree* allows not only to modify any tree-node split but also suggests user other alternative splits that have a high Gain Ratio (Fig. 1D). In this way, we can potentially prevent the tree from blindly pursuing the locally optimal solution, a common issue with *DT*s. In addition, we can instantly see how these changes impact the statistics not only on the training set but also on the test set.

The key element of every *DT* is the splitting rule. In *ITree* we propose three types of algorithms that constitute the tests (Fig. 1A) which can be used individually or together. If the latter option is selected the *ITree* becomes a mixed *DT* in which each split may have different representation. Each test in the internal node can be manually modified (Fig. 1D) by the user in terms representation and its parameters, mainly: in *C4.5* the attribute and threshold; in *TSP* both attributes and in *WTSP* both attributes and the weight factor. Then, by selecting the “Update” button the instances that reached the selected node are rearranged in the following nodes and the statistics for training and testing trees are updated.

### 3.3 Semi-automatic search

To complement the manual construction in the tree classifier, the *ITree* also enables semi-automatic tree building on three levels. The first and most detailed level concerns the choice of a test in an internal node. Here, the user may modify the test in the internal node by entering only some of the information, as shown in Fig. 1D, and leaving the rest of the test parameters empty. For example, if for the *TSP* test only one desired attribute is provided, the second will be found automatically by the *ITree*.

Moving to a higher level, it pertains to the way the chosen test affects the tree. After modifying a split, a user may choose to either update the tree, which can be done using the “Update” button as seen in Fig. 1D, or rebuild the rest of the tree that inherits from the selected node according to the initial settings, achievable with the “Rebuild” button.

The most general level relates to the manner in which a leaf turns into an internal node or subtree. When unfolding the tree, as illustrated in Fig. 1B, the user can opt to expand to a node and two leaves using the “Unfold once” button or create a subtree with the “Unfold all” button. The “Unfold once” option is designed to limit calculations if the user just wants to see how a particular split will divide the data. In addition, the user may choose which algorithm will be used for this operation, which may differ from the initial settings.

### 3.4 Practical validation of *ITree*

In *ITree*, it is straightforward to replicate published tree-solutions with similar representations on user data. It also serves well when a user is looking for a more precise or suitable model based on their needs. To demonstrate *ITree*’s effectiveness in real-life scenarios, we performed a brief validation using a recent publication (Christensen *et al.* 2022) that focused on identifying interactions in omics data for clinical biomarker discovery. Our goal was to check if we could load and use the provided data directly, without any additional steps and at least to some extent achieve similar results.

In the paper, the authors used four datasets, in their original form, from the publication (Christensen *et al.* 2022) GitHub page: proteomics data on Alzheimer’s disease (*AD*) with 137 patients and 1166 protein expressions, gene expression data on diabetes (*OB*) with 46 patients and 200 features, epigenomics data on hepatocellular carcinoma (*HCC*) with 91 patients and 1712 features, and multi-omics data on breast cancer (*BRCA*) with 705 patients and 1937 features.

For the *AD* dataset, it took under 2 min on a regular laptop using the Chrome web browser to build the advanced mixed tree model (with *C4.5* and *TSP* algorithms), draw it, and make it interactive. Similar to the QLattice algorithm from the source publication, the *MAPT* (tau protein) was identified as the most important part of the *ITree*, and the overall model was as simple and competitive as the one in the publication.

For the *OB* dataset, *ITree* instantly generated a tree with perfect classification for the rule: if *PPARGC1B* is smaller than the obesity-related lncRNA *SLC25A21_AS1*, then classify as “not obese,” otherwise “obese.” For the HCC dataset, the *ITree* system also found a tree with a single *TSP* rule in under a minute that could classify 98% of the data correctly. When the *ITree* is using all the available tests (*C4.5, TSP* and *WTSP)* the tree induction time is longer due to *WTSP* complexity.

The last dataset, *BRCA*, was not tested due to its size (over 10 MB), making it cumbersome to use *ITree* as the calculation time might exceed 10 min. Therefore, prior feature selection is advised as all calculations are performed locally in the user’s web browser.

## 4 Conclusion

In this article, we present a new way of classifying with decision tree. The *ITree* tool enables a more user-driven and interactive approach to building the classification tree, allowing users to understand and influence its structure, representation, and divisions of the data. *ITree* enables data exploration through manual, semi-automatic, and automatic approaches, offering users diverse experimental capabilities. In addition, it allows users to observe in real-time how their changes impact the final structure of the tree, as well as their effects on the validation/test datasets. With its ability to import and export decision tree models, ITree enables the saving and reuse of generated structures, even from different sources like scikit-learn. Finally, by adopting the concept of relative expression analysis, which is particularly dedicated to genomic data, *ITree* enables a more thorough exploration of large-scale, multi-dimensional biomedical data. This integration enhances the tool’s ability to handle complex datasets, providing users with deeper insights, especially in the context of biomedical research. It complements other existing solutions, enriching the landscape of *TSP* algorithms and tree-based approaches with its unique features and capabilities, particularly in the application to genomic and other omics data (Afsari *et al.* 2015, Le and Moore 2020, Marzouka and Eriksson 2021).

At the same time, *ITree* serves as an excellent educational tool, making it highly suitable for medical professionals, doctors, and individuals who may not fully understand AI or are not technically inclined. Its user-friendly interface bridges the gap between complex data analysis and practical, everyday application in medical and research fields. Its user-friendly interface bridges the gap between complex data analysis and practical, everyday application in medical and research fields.

Currently, the *ITree* platform is web-based, but we offer the flexibility to download the source code for local deployment, even without an internet connection. Looking ahead, there are plans to introduce a client-server version in which heavy computations will be performed server-side, rather than relying on the user’s browser. This enhancement aims to streamline the user experience and increase the efficiency of data processing.

## Supplementary Material

btae273_Supplementary_Data
