# Supplementary material for: ITree: a user-driven tool for interactive decision-making with classification trees
Source: Bioinformatics. 2024 Apr 18;40(5):btae273. doi: 10.1093/bioinformatics/btae273 (PMC11091738; doi:10.1093/bioinformatics/btae273)
Supplement: btae273_Supplementary_Data [file btae273_supplementary_data.docx]

*Supplement: ITree: a user-driven tool for interactive decision-making with classification trees*

Hubert Sokołowski^1^, Marcin Czajkowski^1*^, Anna Czajkowska^2,3^, Krzysztof Jurczuk^1^ and Marek Kretowski^1^

^1^Faculty of Computer Science, Bialystok University of Technology, Bialystok, Poland,

^2^Department Clinical Research Centre, Medical University of Bialystok, Bialystok, Poland

^3^Department of Medical Biology, Medical University of Bialystok, Poland

This supplement extends the publication by elaborating on the usability and functionality of ITree, while also providing a comprehensive scientific description of the crucial parts of the proposed system.

Contents

[**1.** **Usage** 1](#_Toc161529837)

[**2.** **ITree tests** 2](#_Toc161529838)

1. [***C4.5-like splitting tests*** 2](#_Toc161529839)
2. [***Top-Scoring-Pair (TSP)-like splitting tests*** 3](#_Toc161529840)
3. [***Weight TSP (WTSP)-like splitting tests*** 5](#_Toc161529841)

[**3.** **Reuse of pre-build decision tree model** 6](#_Toc161529842)

1. [**Import and export** 6](#_Toc161529843)
2. [**Load existing tree from scikit-learn** 6](#_Toc161529844)

[**4.** **Technical details** 7](#_Toc161529845)

1. **Usage**

- Initial Configuration
  - 1. File Upload: Begin by uploading your dataset in CSV format, ensuring it aligns with the expected schema for optimal analysis. The example format is as follows.

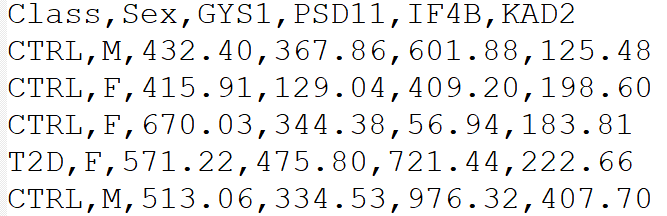


There are no restrictions on which column the 'Class' parameter should be situated.

- - 1. JSON Upload: If you choose to upload a stored decision tree model in JSON format, ITree will use this model to draw the decision tree for the loaded data, bypassing all configuration steps except for decision attribute selection.
    2. Algorithm Selection: Opt from a range of algorithms like C4.5, TSP, and/or WTSP for node splitting, each offering unique strengths depending on your dataset's characteristics.
    3. Decision Attribute Configuration: Carefully select the attribute that will serve as the focal point for classification to guide the decision-making process effectively.
    4. Parameter Settings: Fine-tune the analysis by configuring key settings such as minimum node size, maximum tree depth, and entropy threshold, which are crucial for tailoring the decision tree to your specific research questions.
    5. Draw Tree: Once your configurations are set, initiate the tree generation process by clicking 'Draw,' unveiling the initial structure of your decision tree based on the provided dataset.
- Interactive Decision Tree Viewer

1. Training and Testing Data View: The interface prominently displays the decision tree constructed from your training data, complete with functionality to apply a testing set for assessing the model's predictive accuracy.
2. Tree Node Interaction: Engage with the tree dynamically by clicking on nodes to expand or collapse branches, offering an intuitive means of manual optimization and exploration.
3. Statistics and Metrics: The platform provides instant feedback on various metrics, including accuracy and the confusion matrix, as you manipulate the tree, offering insights into the model's performance.

- Manual Adjustments and Tests

1. Node Modification: Exercise control over your decision tree's structure by manually adjusting splits or selecting different nodes to explore alternative branching strategies.
2. Test Selection: At each node, experiment with various splitting tests (C4.5, TSP, WTSP) to refine how your data is segmented, enabling a granular approach to optimizing classification.

- Real-time Data Analysis

1. Confusion Matrix: Directly access and interpret the confusion matrix within the interface to gauge the precision of your decision tree in classifying data points accurately.
2. Data Viewer: Delve into your dataset's distribution and scrutinize the classification outcomes at each node, facilitating a deeper understanding of the decision tree's logic and effectiveness.

- Experimentation and Learning

1. Interactive Learning: ITree acts as an educational tool, allowing users to experiment with decision tree configurations and learn about their effects on data classification.
2. Export Tree: Users can export the current tree model (excluding data) in JSON format for storage or further analysis.
3. **ITree tests**
   1. ***C4.5-like splitting tests***

The C4.5 algorithm, applied in ITree, is a method of generating a decision tree designed to handle datasets with multiple classes through a series of binary splits. Each split in the tree is determined using entropy-based criteria to measure the impurity or disorder within a set of instances, and information gain to choose the best attribute for splitting the dataset into two subsets. The entropy of a dataset $D$, with multiple classes is defined as:

$$Entropy\left( D \right)= -\sum_{i=1}^{m} p_{i}\log_{2} \left( p_{i} \right).$$

In this formula, $m$ represents the total number of different classes in the dataset, and $p_{i}$ is the proportion of instances belonging to class $i$ in $D$. This entropy formula captures the amount of information or uncertainty present in the entire dataset before any splits.

The objective of the C4.5-like splitting tests is to maximize the information gain, which is the reduction in entropy achieved by partitioning $D$ based on an attribute $A$. The information gain from such a split is calculated as follows:

$$Gain\left( D, A \right)= Entropy\left( D \right)- \left( \left( \frac{\left| D_{1} \right|}{\left| D \right|} \right)Entropy\left( D_{1} \right)+ \left( \frac{\left| D_{2} \right|}{\left| D \right|} \right)Entropy\left( D_{2} \right) \right)$$

Here, $D_{1}$ and $D_{2}$ represent the datasets resulting from the binary split based on attribute $A$, $\left| D \right|$ is the total number of instances in $D$, and $\left| D_{1} \right|$ and $\left| D_{2} \right|$ are the numbers of instances in $D_{1}$ and $D_{2}$, respectively. The goal is to select the attribute $A$ that leads to the highest information gain, effectively reducing uncertainty and improving the clarity of the classification provided by the tree.

For continuous attributes, the C4.5 algorithm within ITree identifies an optimal threshold value that divides the data into two groups, maximizing the information gain. For nominal (discrete) attributes, it evaluates each possible division and chooses the one that results in the highest information gain, using the gain ratio to counteract bias toward attributes with more levels.

This approach allows the decision tree to handle datasets with multiple classes effectively, choosing the best binary splits at each node to progressively increase the predictability and accuracy of the model.


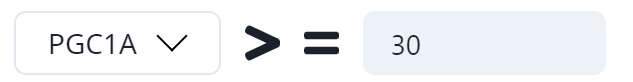
As we select C4.5-like test from induced tree we see that it is in a form of:

$attribute \geq threshold value$ e.g.:

Such tests are called univariate, axis-parallel, or axis-aligned as the decision rule is based on a single attribute compared against a threshold value, leading to a split along one dimension or axis of the data space.

If the condition is holds, then the instances are directed into the left subtree, otherwise to the right. Additional options allows to modify both attribute, threshold, let ITree adjust the threshold for a desired attribute or find top split. User can also view which alternative tests could be used together with their gain ratio score.

- 1. ***Top-Scoring-Pair (TSP)-like splitting tests***

Top Scoring Pair (TSP) classifier was developed which is a straightforward prediction rule utilizing building blocks of rank-altered gene pairs in case and control comparison. Discrimination between two classes depends on finding one pair of genes that achieves the highest-ranking value called "score", which is calculated as follows. The general schema of the TSP algorithm is illustrated in Figure 1.





*Figure 1. A general schema of Top Scoring Pair (TSP) classifier*

Consider a gene expression dataset consisting of $P$genes and$M$samples. Let the data be represented as a $P\times M$ matrix in which an expression value of $i$-th gene from$j$-th sample is denoted as $x_{ij}$. Each row represents an observation of a particular gene over$M$training samples, and each column represents a gene expression instance composed from $P$genes. Let's for the simplicity of presentation assume that there are only two classes: $C_{1}$and $C_{2}$, and instances with indexes from $1$ to $M_{1}$ ($M_{1}<M$) belong to the first class ($C_{1}$) and instances from range $\langle M_{1}+1,M\rangle$ to the second class ($C_{2}$). The discriminating power of each pair of genes $(i,j)$ ($i,j\in\{1,...,P\}$, $i\neq j$) is measured by the absolute difference between the probabilities $p_{ij}$ of the event that gene $i$ is expressed more than gene $j$in the two classes ($x_{im}<x_{jm}$ where $m=1,2,...,M$). For each pair of genes $(i,j)$two probabilities are calculated $p_{ij}(C_{1})$ and $p_{ij}(C_{2})$:

$p_{ij}(C_{1})=\frac{1}{|C_{1}|}\sum_{m=1}^{M_{1}} I(x_{im}<x_{jm})$, $p_{ij}(C_{2})=\frac{1}{|C_{2}|}\sum_{m=M_{1}+1}^{M} I(x_{im}<x_{jm})$,

where $|C_{1}|$denotes the number of instances from class $C_{1}$ and $I(x_{im}<x_{jm})$ is the indicator function defined as:

$I(x_{im}<x_{jm})=\left\{\begin{matrix} 1, if x_{im} < x_{jm} \\ 0, if x_{im} \geq x_{jm} \end{matrix}\right.$.

TSP is a rank-based method, therefore, for each pair of genes $(i,j)$the "score" denoted $\Delta_{ij}$is calculated as: $\Delta_{ij}=|p_{ij}(C_{1})-p_{ij}(C_{2})|$. In the next step, the algorithm chooses a pair with the highest score.

The titled top-scoring pair becomes a pair of genes with the highest score estimated from the training data that in context of ITree reached particular node. In the automatic or semi-automatic mode, the ITree shows divides the data according to the top pair.


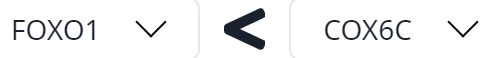
In iTree, a TSP test manifests as a bivariate comparison

$attribute_{1}<attribute_{2}$ e.g.:

directly contrasting two attributes that form the selected gene pair. This comparison serves as a decision checkpoint within the node: if the test's condition is satisfied, ITree leans towards the class or subtree associated with a higher probability in the training dataset; otherwise, it opts for the alternative class/subtree.

This pairwise comparison split, devoid of linear attribute weighting, embodies a binary relational form. Within bioinformatics, its utility is unparalleled, spotlighting nuanced yet vital genomic patterns, like the identification of key markers or "biological switches" indicative of gene expression shifts or regulatory network alterations. The TSP's methodological simplicity, interpretive transparency, and direct relevance to molecular data analysis render it an invaluable asset in uncovering the cryptic mechanisms underlying biological processes.

- 1. ***Weight TSP (WTSP)-like splitting tests***

WTSP-like splitting tests in ITree go beyond the binary classification simplicity of the TSP method by evaluating the relative expression differences between pairs of genes, weighted by a factor that quantifies the strength of their expression relationship. This approach can be symbolically represented as


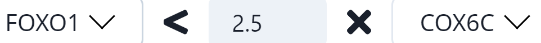
​ $attribute_{1}<weight* attribute_{2}$ e.g.:

where the weight factor adjusts the comparison to identify not only which gene is expressed more but also how much more it is expressed relative to another.

Such weighted comparisons are crucial in bioinformatics applications, where the relative abundance of gene expressions can indicate critical biological processes or disease states. The WTSP test thus offers a more flexible and informative approach to understanding gene interactions, providing insights that are directly applicable to biomarker discovery and the elucidation of gene regulatory networks.

Implementing WTSP-like tests entails considerable computational complexity, as it necessitates evaluating all possible gene pairs across all samples to identify those with the highest discrimination power. Let us consider the same datasets and notion as in TSP search. The discriminating power of each pair of genes $(i,j)$
($i,j\in\{1,...,P\}$, $i\neq j$) in context of WTSP is measured by the highest averaged over instances probability $p$ of an event $\frac{x_{im}}{x_{jm}} < \frac{x_{in}}{x_{jn}} (m\in C_{1} and n\in C_{2}).$ For each pair of genes $(i,j)$, the probability $p_{ij}$ is calculated as follows:

$$p_{ij}=\frac{1}{|C_{1}|\times{|C}_{2}|}\sum_{m=1}^{M_{1}} \sum_{n=M_{1}+1}^{M} \Theta(\frac{x_{im}}{x_{jm}} < \frac{x_{in}}{x_{jn}} )$$

where $|C_{1}|$ denotes the number of instances from class $C_{1}$ and $\Theta(\frac{x_{im}}{x_{jm}} < \frac{x_{in}}{x_{jn}} )$ is the indicator function defined as:

$$\Theta(\frac{x_{im}}{x_{jm}} < \frac{x_{in}}{x_{jn}}) = \left\{ \begin{aligned} 1, if \frac{x_{im}}{x_{jm}} < \frac{x_{in}}{x_{jn}} \\ 0, if \frac{x_{im}}{x_{jm}} \geq\frac{x_{in}}{x_{jn}} \end{aligned} \right.$$

Subsequently, similar to the TSP approach, a "score" $\Delta_{ij}$is computed, and the pair with the highest score is selected to construct the splitting node.

1. **Reuse of pre-build decision tree model**
   1. **Import and export**

ITree system allows import and exporting decision tree model in a JSON format so it can be stored and reused. By choosing +JSON icon, user can load pre-build tree model that will be used on the training data. After rebuilding the tree, user is free to modify its structure alike it was induced from scratch.

User can also export actual state of the decision tree model that appears on the training data to a JSON file. In order to do that, he needs to click Export tree button and the model of decision tree (without the data) will be saved on his hard drive.

An example of the JSON format:


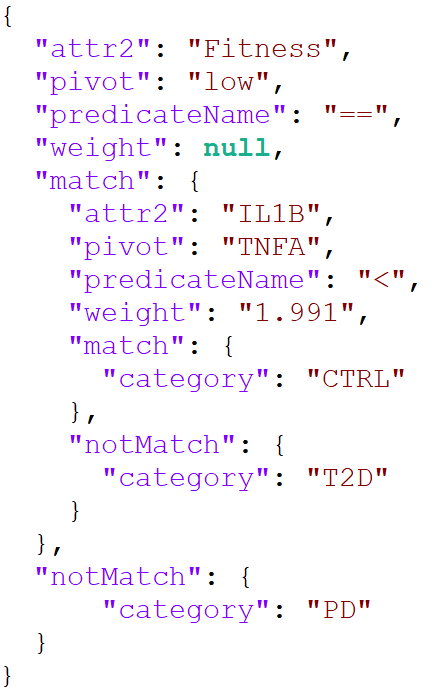


- 1. **Load existing tree from scikit-learn**

mkRecognizing the Python community as a principal user base for decision tree models, we've developed a method for importing trees created using the scikit-learn package into iTree. On our GitHub page, we demonstrate how a concise code snippet can export a tree generated with scikit-learn into a JSON file, formatted for compatibility with iTree. This integration enables Python users to effortlessly load and examine their decision trees in iTree, taking full advantage of the system's extensive features.

For comprehensive guidance, our iTree website (<https://itree.wi.pb.edu.pl>) and GitHub repository (<https://github.com/hsokolowski/iTree>) feature a detailed example using the iris dataset. This tutorial covers the entire process—from loading and training a decision tree classifier in Python's scikit-learn to exporting it in JSON format. Subsequently, the exported JSON file can be utilized within iTree with the same iris dataset, facilitating the examination of the tree structure or the execution of more sophisticated decision splits.

Please note, it's crucial that the attributes in the JSON file, which encapsulate the decision tree model's structure, precisely match the attributes of the data being loaded. This ensures seamless integration and functionality within the iTree system.

1. **Technical details**

- ITree is available at <https://itree.wi.pb.edu.pl>. The source code, documentation and examples are available at our GitHub page <https://github.com/hsokolowski/iTree>.
- Installation and Setup
  - 1. Before installing ITree, ensure you have the following prerequisites installed: Node.js and npm (comes with Node.js)
    2. Clone the Repository
    3. Navigate to the Project Directory and Install Dependencies
- Starting the App

1. To run the app in the development mode
2. Open <http://localhost:3000> to view it in your browser.

- Code Organization - the ITree codebase is structured to facilitate easy navigation and understanding. Key components are organized in separate directories.

1. Available Scripts:
   - - 1. npm start: Runs the app in development mode. Access it at <http://localhost:3000>.
       2. npm test: Launches the test runner in interactive watch mode.
       3. npm run build: Builds the app for production to the build folder. It optimizes the build for performance.
       4. npm run eject: Removes the single build dependency from your project, copying all configuration files and transitive dependencies into your project.
2. Dependencies: ITree utilizes several key libraries and frameworks, including React. The detailed list of dependencies can be found in the package.json file.
